# Supplementary material for: Inhibition of eIF6 Activity Reduces Hepatocellular Carcinoma Growth: An In Vivo and In Vitro Study
Source: Int J Mol Sci. 2022 Jul 13;23(14):7720. doi: 10.3390/ijms23147720 (PMC9319760; doi:10.3390/ijms23147720)
Supplement: Supplementary file 1 [file ijms-23-07720-s001.zip › ijms-1799474-SI.pdf]

Supplementary Information

This file contains Figure S1 and Figure S2.

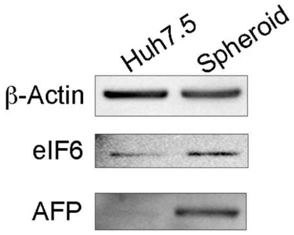

Figure S1. eIF6 and AFP protein levels increase in HCC spheroids.

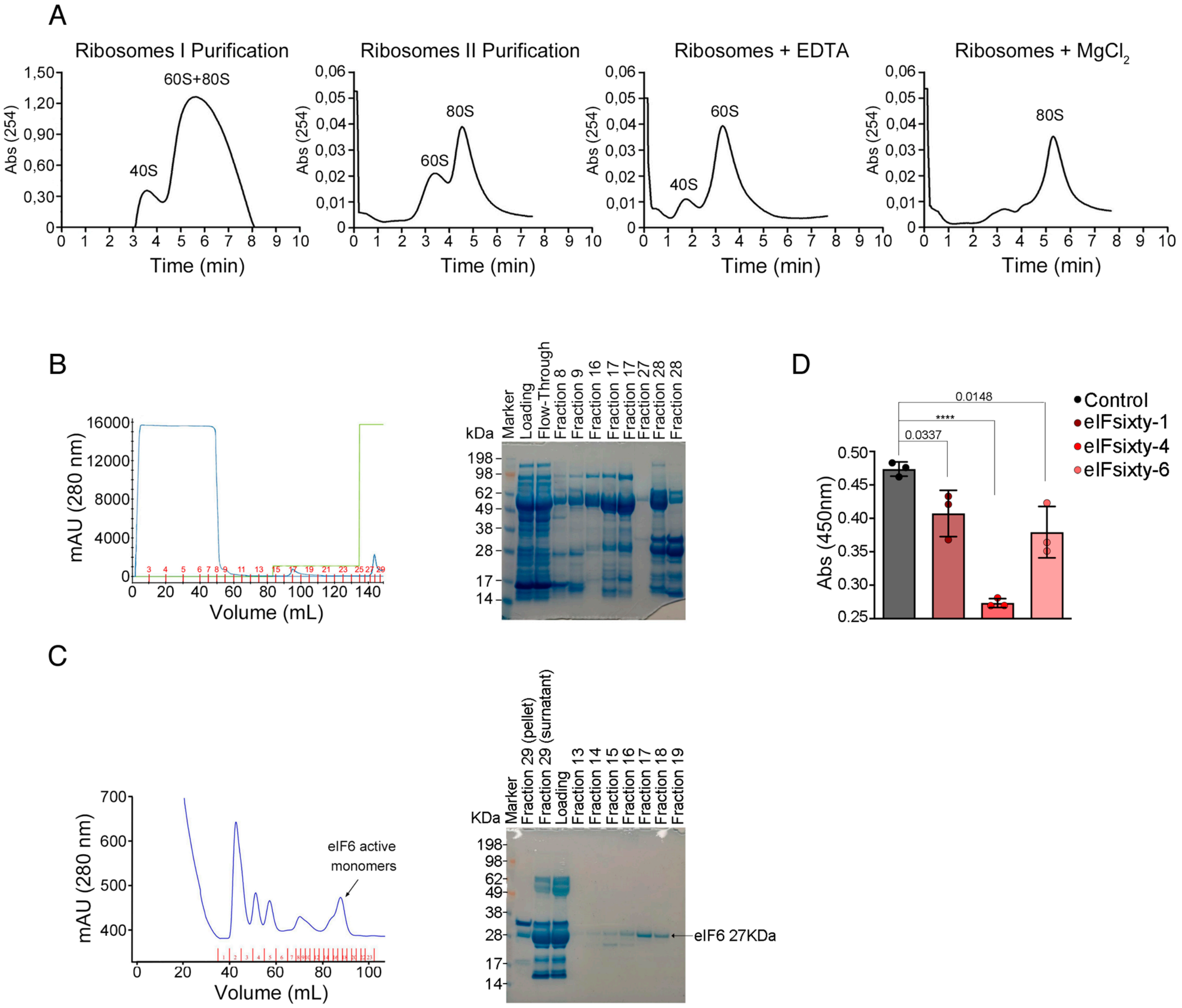

Figure S2. Ribosomes and recombinant eIF6 protein purification. Assay on pure 60S Arthemia ribosomes (A) Ribosomes purifications from Arthemia Salina eggs are represented in the first and the second profile. 80S monosomes were dissociated into 40S and 60S ribosome subunits after EDTA treatment. The addition of $MgCl_2$ allowed joining of 40S and 60S ribosomes. (**B)** His Trap affinity chromatography purification of eIF6 recombinant protein (left) and Blue Coomassie staining of collected fractions (right). (**C)** Purification of eIF6 active monomers: eluted proteins (from B), corresponding to the 28th fraction, were loaded in a gel filtration column: eIF6 monomers (17th and 18th fractions) were eluted, loaded in a 10% polyacrylamide gel and stained with Blue Coomassie. (**D)** iRIA: addition of eIFsixty-i compounds to the reactions impairs eIF6-60S binding. The following concentration are used: 1,4 µM for eIFsixty-1; 5 µM for eIFsixty-4 and 1 µM for eIFsixty-6. One representative of three independent experiments is shown. Two-tailed *t* test, **** *p* value ≤ 0.0001.
